# *BRAF* Mutations Occur Infrequently in Ovarian Cancer but Suggest Responsiveness to BRAF and MEK Inhibition

**DOI:** 10.1200/PO.18.00025

**Published:** 2018-06-14

**Authors:** Maira P. Campos, Melissa Cohen, Erika Von Euw, Victor Velculescu, Jennifer L. Kujak, Dylan Conklin, Dorothy Hallberg, Dennis J. Slamon, Julia Elvin, Gottfried E. Konecny

**Affiliations:** **Maira P. Campos**, **Melissa Cohen**, **Dennis J. Slamon**, and **Gottfried E. Konecny**, David Geffen School of Medicine, University of California, Los Angeles, Los Angeles; **Maira P. Campos**, **Erika Von Euw**, **Dylan Conklin**, **Dennis J. Slamon**, and **Gottfried E. Konecny**, University of California, Los Angeles Translational Oncology Research Laboratory, University of California, Los Angeles, Santa Monica; **Jennifer L. Kujak**, Rolling Oaks Radiology, Thousand Oaks, CA; **Victor Velculescu** and **Dorothy Hallberg**, Sidney Kimmel Comprehensive Cancer Center, Johns Hopkins University School of Medicine, Baltimore, MD; and **Julia Elvin**, Foundation Medicine, Cambridge, MA.

## INTRODUCTION

**Fig 1.**
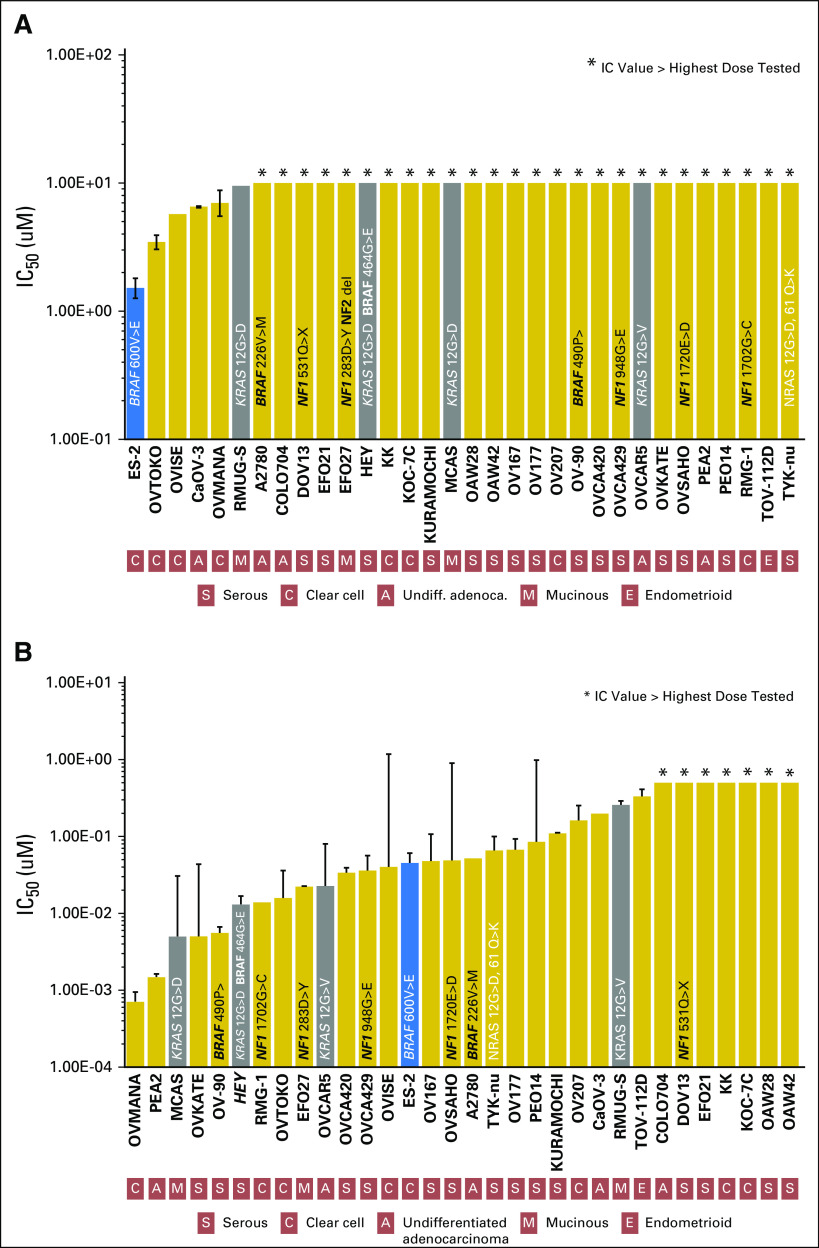
Preclinical activity of (A) vemurafenib and (B) trametinib across a large panel of 32 ovarian cancer (OC) cell lines characterized for *BRAF*, *KRAS*, and *NF1* mutations. Growth inhibition was calculated as a function of the number of generations inhibited in the presence of vemurafenib or trametinib versus the number of generations over the same time course in the absence of the drug as reported previously.^7^ In brief, the log of the fractional growth inhibition was plotted against the log of the drug concentration, and the half-maximal inhibitory concentration (IC_50_) values were interpolated from the resulting linear regression curve fit. Cell lines are ordered left to right according to increasing IC_50_ values. Error bars indicate the SE of the mean value. Mean is derived from at least three replicate experiments. Colored bars denote cell lines with a *BRAF*^V600^ (blue) and *KRAS* (gray) short gene variations. The OC cell line ES-2, which contains a *BRAF*^V600E^ mutation, was the most sensitive to the BRAF inhibitor vemurafenib (A). MEK inhibition with trametinib led to significant growth inhibition in the ES-2 ovarian cells but also in other cell lines harboring *KRAS*, *NF1*, and other *BRAF* mutations.

Ovarian cancer (OC) is the leading cause of death from gynecologic malignancies in the developed world.^1^ Although substantial efforts have been devoted to identifying potentially actionable oncogenic driver mutations in OC, only a few genes are frequently mutated, particularly in high-grade serous OC (HGSOC), the most common histologic subtype. The Cancer Genome Atlas reported *p53* mutations in almost all primary HGSOC tumors (96%) but a low prevalence of recurrent somatic mutations in additional genes including *BRCA1* (12%), *BRCA2* (11%), *NF1* (4%), and *CDK12* (3%).^2^ In the COSMIC database, additional infrequent mutations such as *KRAS* (6%), *PIK3CA* (2%), and *BRAF* (2%) have been identified in HGSOC.^3^ We believe that some of these mutations, although rare, are important drivers in HGSOC. For example, mutations in *BRAF* lead to constitutive activation of downstream *MEK1* and *MEK2* and may regulate proliferation and survival in ovarian tumor cells as in many other cancers.^4^ The most commonly observed *BRAF* mutation, V600E, accounts for 90% of the *BRAF* mutations found in all patients with cancer.^3^ Treatment with BRAF inhibitors such as vemurafenib or dabrafenib in patients with advanced *BRAF*^V600^-mutated melanoma has shown objective tumor responses in approximately half of the patients.^5^ Recent clinical studies have suggested that concurrent inhibition of the BRAF and MEK kinases of the mitogen-activated protein kinase (MAPK) pathway with trametinib or cobimetinib can decrease MAPK-driven acquired resistance, resulting in greater efficacy and a decrease in the cutaneous toxicities observed from paradoxical MAPK pathway activation with BRAF inhibitor monotherapy.^6^ Here, we report a patient with recurrent HGSOC who had experienced treatment failure with an extensive number of prior treatment regimens and whose tumor was found to harbor a *BRAF*^V600E^ mutation. Of note, this patient responded extremely well to treatment with a BRAF inhibitor (vemurafenib) and subsequently to a BRAF inhibitor in combination with a MEK inhibitor (dabrafenib and trametinib). To further underscore this treatment rationale, we studied the growth-inhibitory effects of the BRAF inhibitor vemurafenib and the MEK inhibitor trametinib across a panel of 32 OC cell lines that were each characterized for mutational status of *BRAF*, *KRAS*, and *NF1* (Fig 1). Cell lines, assays, and sequencing methods have been described earlier.^7^ In brief, cells were plated into 24-well tissue culture plates and grown with or without increasing concentrations of inhibitors. Cells were counted on days 1 and 6 using a Coulter Z2 particle counter (Beckman Coulter, Indianapolis, IN). Growth inhibition was calculated as a function of the number of generations inhibited by vemurafenib or trametinib.

## INDEX PATIENT

**Fig 2.**
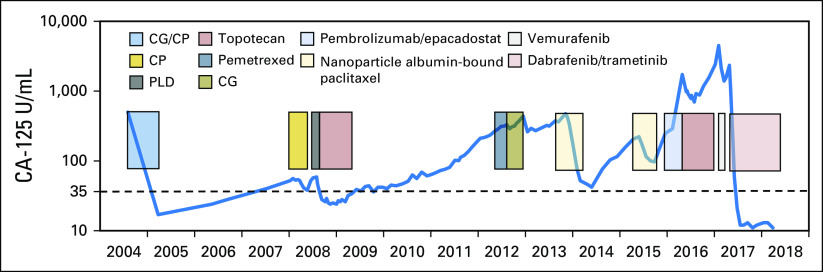
Diagram outlining the entire treatment history with corresponding CA-125 levels. CP, carboplatin and paclitaxel; GP, carboplatin and gemcitabine; PLD, liposomal doxorubicin.

**Fig 3.**
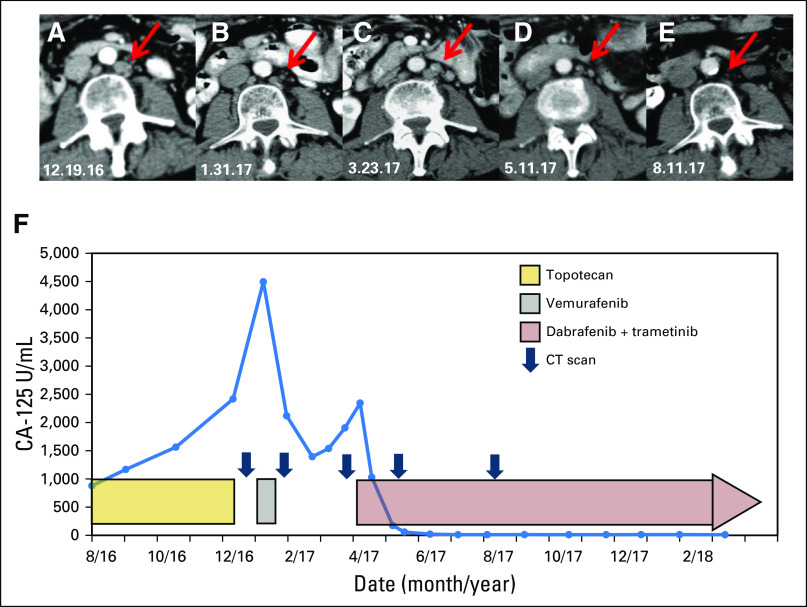
Computed tomography (CT) scans of the abdomen and pelvis revealed a partial response after a brief treatment with vemurafenib. For example, a left periaortic lymph node is (A) initially seen measuring 11 × 14 mm at baseline and (B) subsequently decreased to 7 × 8 mm after brief treatment with vemurafenib. (C) After holding vemurafenib, CT scans of the abdomen and pelvis showed renewed progression of the lymphadenopathy with the left periaortic node measuring 10 × 11 mm. (D) Dabrafenib and trametinib treatment again led to a significant decrease in size of the abdominal and retroperitoneal lesions with a decrease in size of the left periaortic node to 4 × 6 mm. (E) After further combination therapy, the CA-125 level remains in the normal range and updated CT scans demonstrate an ongoing partial response with continued reduction in size of the patient’s left periaortic lymph node now measuring 2 × 3 mm. (F) CA-125 tumor marker levels are shown during treatment while on chemotherapy, followed by the BRAF inhibitor vemurafenib given as a single agent, followed by combination treatment with the BRAF inhibitor dabrafenib and the MEK inhibitor trametinib.

A 70-year-old woman presented with recurrent *BRCA* wild-type HGSOC. Fourteen years before presentation, she had optimal cytoreductive surgery for International Federation of Gynecology and Obstetrics stage IIIC HGSOC followed by front-line chemotherapy with carboplatin plus gemcitabine and carboplatin plus paclitaxel. She developed recurrent disease to the left paracolic gutter region, which was not amenable to surgery but treated with carboplatin plus paclitaxel and, after further progression, by liposomal doxorubicin. Subsequent treatments included topotecan, pemetrexed, gemcitabine plus carboplatin, weekly nanoparticle albumin-bound paclitaxel, and then pembrolizumab in combination with epacadostat followed by topotecan plus bevacizumab (Fig 2). Upon further progression, a computed tomography (CT)–guided core biopsy was performed on an external iliac lymph node. In the absence of a therapeutic standard of care, tumor tissue was submitted to Foundation Medicine for comprehensive genomic profiling revealing an activating *BRAF*^V600E^ mutation as well as *ASXL1* (K580fs*2) and *RUNX1T1* (R520H) mutations. In preclinical studies, ES-2 cells that harbored a *BRAF*^V600E^ mutation were the most sensitive to vemurafenib (Fig 1A). Antiproliferative effects of trametinib varied significantly between individual cell lines, with up to a 3-log-fold difference in the half-maximal inhibitory concentration values, but were most pronounced in cell lines that harbored *BRAF*, *KRAS*, or *NF1* mutations (Fig 1B). After providing informed consent, the patient was treated in accordance with the declaration of Helsinki with the BRAF inhibitor vemurafenib at the US Food and Drug Administration–approved dose of 960 mg twice a day. Because of fear of adverse effects, the patient only took half of the prescribed dose of 480 mg twice daily. Despite the dose reduction, she had to stop treatment after 13 days as a result of a profound diffuse maculopapular skin rash, which significantly improved with systemic corticosteroid medication. Despite the brevity of treatment, the patient’s CA-125 levels decreased from 4,495 to 2,119 U/mL within 21 days of initiating therapy, and a CT scan of the abdomen and pelvis revealed a partial response (PR) 29 days after commencing vemurafenib therapy.^3^ While the patient remained off therapy awaiting insurance approval for dabrafenib plus trametinib, the CA-125 level increased again from 1,394 to 2,345 U/mL with increasing symptoms of abdominal bloating and distension as well as moderate pelvic pain. CT scans of the abdomen and pelvis done before starting new treatment showed renewed progression (Fig 3). After starting combination therapy with dabrafenib (150 mg twice a day) and trametinib (2 mg daily), the CA-125 levels decreased from 2,345 to 12 U/mL (normal range, < 35 U/mL) within a 10-week period. The patient tolerated medication well without relevant skin toxicity. A CT scan of the abdomen and pelvis showed pronounced decrease in size of the abdominal and retroperitoneal lesions consistent with a radiographic PR. In the following weeks, the patient developed mild lower extremity edema and moderate hypertension, requiring a decrease in the doses of dabrafenib from 150 mg to 75 mg twice a day and of trametinib from 2 mg to 1 mg daily. After 10 months of combination therapy, the CA-125 levels remained in the normal range, and updated CT scans demonstrated an ongoing PR with continued reduction in size of her retroperitoneal lymphadenopathy (Fig 2). To identify additional patients who might benefit from BRAF/MEK inhibition, we analyzed a subset of 2,983 ovarian tumors from the Foundation Medicine database of more than 125,000 clinical cases that had undergone hybrid capture–based next-generation sequencing. *BRAF*^V600^ mutations were found in 46 (1.5%) of 2,983 serous OCs, in three (4.2%) of 71 ovarian mucinous cancers, in one (3.6%) of 28 OCs with neuroendocrine differentiation, in four (5%) of 80 low-grade serous OCs, and in two (13.3%) of 15 serous borderline tumors (Table 1).

**Table 1.**
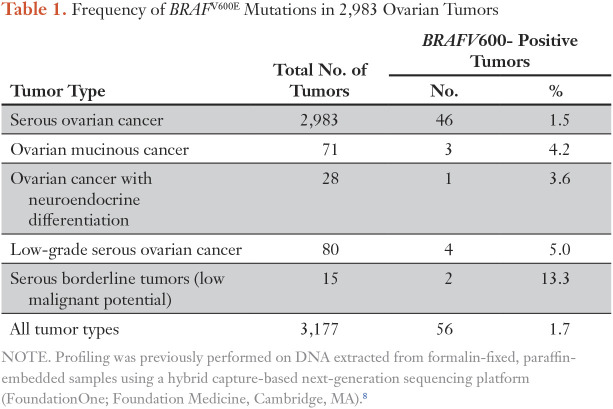
Frequency of *BRAF*^V600E^ Mutations in 2,983 Ovarian Tumors

## DISCUSSION

We present a patient diagnosed with recurrent HGSOC who had received an extensive number of prior chemotherapy regimens and whose tumor was found to harbor a *BRAF*^V600E^ mutation. Treatment with the BRAF inhibitor vemurafenib and subsequently the BRAF inhibitor dabrafenib and MEK inhibitor trametinib led to a persistent radiographic PR with normalization of CA-125 and marked symptomatic improvement. This is a remarkable response, especially in light of the patient’s extensive prior treatment and the absence of a meaningful alternative treatment option. The rapid development and widespread availability of NGS provides the opportunity to select an appropriate targeted therapy.^9^ However, matching patients with the optimum therapy is often challenging, particularly as driver events are generally infrequent and comprehensive clinical response data are rarely available for the specific cancer type, especially with noncanonical variants. Because a conventional clinical trial design approach may not be feasible to demonstrate the effectiveness of a targeted agent in rare molecular events in a given cancer type, basket trials have been initiated that categorize patients’ cancers on the basis of the sequencing of the tumor, rather than the organ of origin.^10^ Nevertheless, individual case reports, such as the present description, may provide important additional evidence to support further use of a BRAF/MEK inhibitor combination in OC found to harbor a *BRAF*^V600^ mutation, which has been shown to be a molecular driver in a meaningful subset of other cancers, including malignant melanoma, papillary thyroid carcinomas, colorectal cancers, and non–small-cell lung cancers.^11^ In our OC cell line panel, ES-2 cells,^12^ which contain a *BRAF*^V600E^ mutation, were the most sensitive to the BRAF inhibitor vemurafenib. Similarly, MEK inhibition with trametinib led to growth inhibition in ES-2 cells; however, OC cell lines with *KRAS*, *NF1*, and other *BRAF* mutations also seemed to be sensitive to MEK inhibition, suggesting potentially a broader role of MEK inhibitors in OC. However, the sensitivities of these OC cell lines differ from what is observed in melanoma cell lines, for which these inhibitors are a standard of care. For sensitive melanoma cell lines, the typical half-maximal inhibitory concentrations to vemurafenib or trametinib are less than 300 nM or in the low nanomolar range, respectively.^13^

Although it is clear that NGS-based technologies are already having an impact on patient care, for broad, widespread use to occur, several factors will need to be addressed in the coming years. Funding organizations must embrace the need for more robust predictive tumor models and new cancer research tools and assist in their development. Examples include establishing larger cancer cell line collections with integrated molecular characterization. In addition to expanding basket-type trials, we need further development of data-sharing consortia that are focused on generating an evidence base for precision cancer medicine by integrating robustly validated, broad-based genomic profiling data with clinical outcome data. This effort will help generate the scientific evidence needed to further accelerate the adoption of precision medicine into clinical practice in oncology.^14^ However, until further evidence from biomarker-driven clinical trials, observational databases, and improved preclinical identification of genomic determinants of response to therapy are available, case reports such as this one may help guide treatment decisions in individual patients with rare molecular genomic alterations.
